# Indigenous plant growth-promoting rhizobacteria for cocoa growth enhancement and *Phytophthora* pod rot management in coastal Andhra Pradesh, India

**DOI:** 10.1186/s12870-025-07166-6

**Published:** 2025-09-30

**Authors:** B. Neeraja, V. Prasanna Kumari, K. Gopal, G. S. Madhu, K. Vijay Kumar, T. Madhumathi, N.B.V. Chalapathi Rao, B. R. Sayiprathap

**Affiliations:** 1 Acharya N.G. Ranga Agricultural University, Guntur, Andhra Pradesh 522 034 India; 2 Dr. YSR Horticultural University, Eluru, Andhra Pradesh 534 101 India; 3 Indian Institute of Horticultural Research, Bengaluru, Karnataka 560 089 India; 4 World Vegetable Center, South Central Asia, Hyderabad, Telangana 502 324 India

**Keywords:** Cocoa, PGPR, Biocontrol, 16S rRNA, Antagonism, Disease suppression

## Abstract

Plant growth-promoting rhizobacteria (PGPR) are beneficial microbes that support plant development through mechanisms such as nutrient solubilization, phytohormone production, and antagonism against phytopathogens. This study focused on isolating and characterizing PGPR strains native to the cocoa rhizosphere in the coastal region of Andhra Pradesh and assessing their efficacy against *Phytophthora palmivora*, the causal agent of cocoa pod rot. A total of 50 bacterial isolates were initially screened based on hydrolytic enzyme production, siderophore activity, phosphate solubilization, and antagonistic potential. Six promising isolates NEG27, NWG12, NSK3, NEG14, NEG16, and NEG3—showed strong antifungal activity and were identified through 16 S rRNA sequencing as *Pseudomonas fluorescens* (NEG27, NEG3), *Stutzerimonas stutzeri* (NWG12), and *Pseudomonas otitidis* (NEG14, NEG16, NSK3). In vitro antagonism assays demonstrated that NEG27 inhibited *P. palmivora* mycelial growth by 82.26%, followed by NWG12 (77.41%) and NEG14 (76.30%), with microscopy revealing severe hyphal disintegration and malformed zoospores. PGPR seed treatment significantly enhanced cocoa germination rates (35.71% increase over control), seedling vigour index (by 441.84%), and root–shoot biomass (*p* < 0.05). Field trials conducted over two cropping seasons (2022-23 and 2023-24) revealed that combined foliar application of NEG27, NWG12, and NSK3 at 1.5% significantly (*p* < 0.05) reduced pod rot disease severity compared to untreated controls, while also improving yield-related traits and the benefit-to-cost (B: C) ratio. These results highlight the potential of indigenous PGPR strains as sustainable biocontrol agents and viable alternatives to chemical fungicides.

## Introduction

Cocoa (*Theobroma cacao* (L.)) often referred to as the ‘food of the gods’, is an important perennial tropical crop that originated in the rainforests of South America [1]. Cocoa is valued primarily for its beans, which are used in chocolate production, cocoa has become a key economic and agricultural commodity worldwide. Cocoa is a rich source of lipids (50–57%) in addition to proteins (10%), fibre (12%) and carbohydrates (8%). Additionally, it is a good source of essential minerals such as phosphorus, magnesium, iron, zinc, manganese, copper, potassium, and selenium, as well as vitamins B2 and B3, which play vital roles in growth and development [1]. Globally, cocoa is cultivated on 12 million hectares (mha), with an annual production of 5.8 million tonnes (MT) of cocoa beans, yielding an average productivity of 492 kg/ha (FAOSTAT, 2022) supporting over 6 million farmers and serves as a livelihood source for more than 40 million people [2, 3]. Low cocoa yields are attributed to various biotic and abiotic constraints, with pod rot caused by *Phytophthora sp.* being the major biotic constraint affecting cocoa production worldwide. An estimated 30% yield loss, equal to approximately $ 3.8 billion USD, occured due to pod rot diseases alone [4]. Crop productivity and health are closely tied to the soil environment, particularly the rhizosphere. This unique zone around the plant roots is a host for diverse microbiome, where beneficial interactions between plants and microbes play a crucial role in plant health and development [5].

Phytophthora pod rot, commonly known as black pod disease, is a major threat to cocoa cultivation and is primarily caused by the oomycete pathogens *Phytophthora palmivora*, *P. tropicalis*, and *Phytophthora megakarya*. Among these, *P. palmivora* is the most widely distributed and poses a particularly serious threat. *Phytophthora* spp., particularly *P. palmivora*, causes black pod disease worldwide, resulting in 20–30% pod losses and up to 10% annual tree mortality [6, 7]. These pathogens infect cocoa pods through natural openings or wounds, especially under humid and rainy conditions, using sporangia and motile zoospores for infection and dissemination. Once inside host tissues, they disrupt cellular processes and cause necrosis, leading to rapid pod decay. Yield losses typically range from 20 to 30%, but can exceed 90% during severe outbreaks [8]. Effective management relies on integrated disease management (IDM) strategies, including cultural practices such as regular pruning, removal of infected pods, and improved field drainage to reduce pathogen spread (Guest). Chemical control with copper-based fungicides and phosphonates remains common; however, issues like pathogen resistance and environmental concerns necessitate careful, rotational use [8]. Biological control agents such as *Trichoderma* spp. and *Pseudomonas* have shown promise in suppressing *Phytophthora* and enhancing plant defense responses [9, 10]. Given the drawbacks of chemical fungicides, there is an urgent need to develop indigenous plant growth-promoting rhizobacteria (PGPR) that not only support cocoa plant growth but also effectively suppress *Phytophthora*. Moreover, combining compatible PGPR strains may offer synergistic effects, contributing to a more sustainable and efficient disease management approach.

Plant growth-promoting rhizobacteria (PGPR) are soil bacteria that inhabit the plant rhizosphere and promote plant growth and development through various mechanisms. They release phytohormones or other biologically active substances, alter endogenous phytohormone levels, enhance nutrient availability and uptake through fixation and mobilization and reduce the harmful effects of pathogenic microorganisms on plants [5, 11, 12]. PGPR have significant potential applications in sustainable agriculture, especially for long-duration plantation crops such as coconut, arecanut, cocoa, tea, coffee, spices and rubber [13–16]. *Pseudomonas* spp. is commonly used as model organisms for root-colonizing bacteria [17]. Rhizobacteria like *P. fluorescens* and *Bacillus subtilis* have been shown to effectively inhibit the growth of *P. palmivora*, the fungus responsible for cocoa fruit rot [18].

Fungicides, including copper-based products and systemic agents such as metalaxyl, are widely used to manage black pod disease in cocoa. These chemicals are effective in reducing disease incidence and enhancing yields [19, 20] however, over-application can lead to environmental pollution and deteriorate soil health [21]. In light of these limitations, the identification and characterization of plant growth-promoting rhizobacteria (PGPR) from the cocoa rhizosphere has gained importance. Certain PGPR strains, such as *P. fluorescens* and *B. subtilis*, along with fungal biocontrol agents like *Trichoderma* spp., have demonstrated efficacy in managing *Phytophthora* pod rot. These microbes suppress disease by producing antimicrobial compounds, enhancing plant defense responses, and competing with pathogens for space and nutrients [22, 23]. Moreover, the development of bioformulations combining multiple biocontrol agents has further improved their effectiveness. These biological strategies not only help control disease but also promote healthier, more resilient plant growth [24]. Accordingly, the objectives of this study were to: (i) isolate and screen bacterial strains from the cocoa rhizosphere in coastal Andhra Pradesh for plant growth-promoting traits; (ii) identify promising isolates using 16 S rRNA gene sequencing; and (iii) evaluate their efficacy against *P. palmivora* through both in vitro assays and field trials.

## Materials and methods

### Isolation of rhizosphere bacteria

Rhizosphere soil samples (30 cm depth) were collected from 210 plantations across three major cocoa growing districts in costal Anhdra Prdesh by gently uprooting the side roots and shaking off loosely adhering soil. Approximately 5–10 g of soil tightly attached to the roots was carefully brushed off into sterile plastic bags and transported to the laboratory under cool conditions for immediate processing. One gram of rhizosphere soil was suspended in 9 mL of sterile distilled water and serially diluted (1 × 10^−6^, 1 × 10^−7^, and 1 × 10 ^−8^). From appropriate dilutions, 100 µL aliquots were plated onto nutrient agar (NA) plates and incubated for two days at 28 ± 2 °C. Bacterial colonies were then selected for further purification and preserved temporarily in a 20% glycerol solution at − 20 °C.

### Isolation of *Phytophthora *and pathogenicity testing

Pod rot infected sample collected from Avidi village (16° 40’ 25.68” N, 81° 54’ 15.12” E), East Godavari district of Andhra Pradesh in India was used to isolate *Phytophthora* on modified corn meal agar (CMA) [25]. Three *Phytophthora* isolates were obtained from pod rot infected samples, and morphological traits such as colony characteristics, sporangia, and chlamydospores were recorded and compared with the IDPhy database [26] for identification of *Phytophthora* sp. To test pathogenicity, seeds of the cultivated hybrid ‘VTLCH-2’ (Vittal Cocoa Hybrid 2), developed and authenticated by the Indian Council of Agricultural Research (ICAR)- Central Plantation Crops Research Institute (CPCRI), Kasaragod, Kerala, India, were procured under institutional collaboration. Pathogenicity assays were conducted using the agar disc inoculation technique as described by Delgadillo-Duran et al. [27]. The cocoa pods were inoculated with 5 mm mycelial disc by placing in the centre of the cocoa pod, where small holes were made, and then covered with polythene film. The pods were incubated under dark in a BOD incubator (REMI) with 100% humidity at 25^°^C. The expression of symptoms Began 24 h after inoculation. Observations on pod rot lesion development were recorded at 24-hour intervals for up to 4 days post-inoculation.

### DNA isolation and PCR amplification

Six potential bacterial isolates (NEG27, NEG3, NEG16, NEG14, NSK3 and NWG12) selected based on their antagonistic effect against *Phytophthora* in dual-culture plate assay were used for molecular studies. Briefly 1 mL of bacterial culture was used to isolate total genomic DNA using Macherey-Nagel (MN) NucleoSpin Tissue kit by following the manufacturer’s instructions. The DNA quality was assessed by both in 0.8% agarose gel and by using a spectrophotometer (NanoDrop 8000, Thermo Fisher Scientific) and stored in a refrigerator at − 20°C. Synthetic oligonucleotide primers (27F 5’- AGAGTTTGATCCTGGCTCAG-3’ and 1492R 5’-CGGTTACCTTGTTACGACTT-3’) corresponding to 16S rRNA gene was used for amplification. 2 µl template DNA (50 ng/µL) was added to 12.5 µl 2X EmeraldAmp GT PCR Master Mix (Code No. RR310A), 0.2 µM of each specific primer, final volume make up to 25 µl with dH_2_O. The PCR mixture was incubated in a thermocycler (Mastercycler X50a, Eppendorf) by 1 cycle of denaturation at 94 °C for 3 min, followed by 35 cycles at 94 °C for 30 sec, 55 °C for 30 sec, and 72 °C for 1 min. The final extension was at 72 °C for 7 min. Similarly, total DNA was isolated from 100 mg of fungal mat of one representative isolate of *Phytophthora* using the MN kit, following the manufacturer’s instructions. DNA quality was assessed using the same methods as described for bacterial DNA. For *Phytophthora* amplification, oligonucleotide primers corresponding to ITS (ITS1 5’-TCCGTAGGTGAACCTGCGG-3’ and ITS4 5’-TCCTCCGCTTATTGATATGC-3’), β-tubulin (5’-GCCAAGTTCTGGGAGGTCATC-3’ and 5’-CCTGGTACTGCTGGTACTCAG-3’), and mitochondrial cytochrome c oxidase subunit II (COX-II) (FMphy8Bf 5’-AAAAGAGAAGGTGTTTTTTATGG–3’) and FMphy10bR (5’-GCAAAAGCACTAAAAATTAAATATAA-3’) regions were used for the detection. The PCR composition and cycling conditions followed similar to bacterial detection with adjustments in annealing temperatures as per the target gene

### Sequencing and phylogenetic analysis

The PCR products were resolved on a 1.2% agarose gel in TAE buffer and visualized using a gel documentation system (Major Science Image Analyzer). The target amplicons were purified using the MN NucleoSpin^®^ Gel and PCR Clean-up Kit following the manufacturer’s protocol, and subsequently sequenced bidirectionally using the Sanger sequencing method (Eurofins Genomics, India). The raw sequences were aligned and edited using CLC Genomics Workbench. Trimmed sequences were subjected to BLASTn analysis using the NCBI Basic Local Alignment Search Tool to identify the closest matching sequences in the GenBank database. Multiple sequence alignment was carried out using the MUSCLE algorithm. A phylogenetic tree was constructed in MEGA 11 using the Maximum Likelihood (ML), with 1000 bootstrap replications [28].

### Biochemical characterization of bacterial isolates

A series of biochemical tests were conducted to characterize the bacterial isolates based on the criteria of Bergey’s Manual of Systematic Bacteriology [29]. Turbidity away from the line of inoculation was considered a positive indicator of motility. Catalase and oxidase tests were performed according to Taylor et al., [30] and Jurtshuk et al., [31], respectively. To assess the suitability to aerobic or anaerobic environments, the citrate test was conducted using Simmons citrate agar medium [32]. To assess qualitative Indole-3-Acetic Acid (IAA) production, bacterial isolates cultured on Luria-Bertani (LB) agar plates supplemented with 0.1% L-tryptophan at room temperature for 48–72 h were overlaid with Salkowski reagent (2% 0.5 M FeCl₃ in 35% perchloric acid) for 30 min. The presence of IAA was indicated by the development of a pink to red color around the colonies [33]. For ammonia production, bacterial isolates were inoculated into 10 mL of peptone water and incubated at 28 ± 2 °C for 48–72 h. After incubation, 0.5 mL of Nessler’s reagent was added to each tube. The development of a yellow to brown coloration indicated positive ammonia production. The intensity of color was visually assessed and scored qualitatively [34]. Siderophore production was tested by spot inoculating bacterial isolates on Petri dishes containing Chrome Azurol S (CAS) agar, which incorporates CAS dye and an iron (III)-hexadecyltrimethylammonium bromide complex into the agar for 48–72 h. The presence of an orange halo around the colonies indicated siderophore production [35]. Phosphate solubilization was tested by spot inoculating bacterial isolates on Pikovskaya’s agar medium, which contains insoluble tricalcium phosphate as the phosphorus source for 5–7 days. Clear halos around the bacterial colonies indicated phosphate solubilisation [36]. The biofilm formation assay was conducted following the protocol described by O’Toole [37]. Bacterial biofilms were grown in 96-well plates using diluted overnight cultures in M63 minimal medium, incubated at 37 °C for 4–24 h, stained with 0.1% crystal violet, washed, dried, and solubilized with 30% acetic acid. Biofilm biomass was quantified at 550 nm. All the tests were carried in three replications for each isolate.

### *In vitro* testing of bacterial isolates for their antagonism in dual-culture method

Dual culture plate method was used for the study. Fresh pathogen (mycelial disc) and bacteria (streaking) were inoculated 2.5 cm apart on the same after PDA plate. Per cent inhibition of *P. palmivora* was calculated by predefined formula given below. 

$$\mathrm I\;=\;\frac{\displaystyle\mathrm C-\;\mathrm T}{\mathrm C}\;\times100$$

Where, 

 I= Per cent inhibition over the control 

 C = Growth of the pathogen in control plate

T = Growth of the pathogen in dual cultured plate.

Morphologies of hyphae in the vicinity of bacterial colonies were observed under a phase-contrast microscope (Olympus BX-53, Germany) and images were recorded. Each set was replicated three times.

### Effect of bacterial seed treatment on cocoa seed germination and vigour

Bacterial cells were harvested from 2–3-day-old cultures grown in nutrient broth (NB) medium by centrifugation at 15,000 rpm for 1 min at 4 °C. The pellet was resuspended in 0.6 ml of sterile distilled water to obtain a final concentration of 2 × 10⁸ CFU/ml for seed treatment. Cocoa seeds (variety: VTLCH-2) were surface sterilized using 1% sodium hypochlorite (NaOCl) for 1 min, followed by three washes in sterile distilled water. The sterilized seeds were then inoculated with suspensions of six different plant growth-promoting rhizobacteria (PGPR) isolates and air-dried overnight at room temperature under sterile conditions. A total of 20 seeds per treatment were sown in pots under controlled conditions. Seeds without PGPR treatment were maintained as negative controls to assess the impact of bacterial inoculation on germination and seedling vigour. Each set was replicated 4 times. The germination percentage, root and shoot length of the plants and seedling vigour index were calculated by using the formula,

$$\mathrm{Germination}\;\;\mathrm{percentage}\;(\%)\;=\frac{\mathrm{Number}\;\mathrm{of}\;\mathrm{seeds}\;\;\mathrm{germinated}}{\mathrm{Total}\;\mathrm{number}\;\mathrm{of}\;\mathrm{seeds}}\;\times\;100$$

Vigour index = Germination (%) x (Mean shoot length + Mean root length).

### Field studies: effect of PGPR foliar spray on disease severity and yield

A field experiment was conducted during the 2022/2023 and 2023/2024 rainy seasons in a farmer’s field located in Avidi Village (16°40’25.68” N, 81°54’15.12” E), East Godavari district, Andhra Pradesh, India, using a locally cultivated cocoa variety of the Forastero type. The study was carried out with prior consent and in accordance with the guidelines laid down by Dr. Y.S.R. Horticultural University, Andhra Pradesh. A total of 23 treatments were evaluated to determine the efficacy of PGPR isolates in managing Phytophthora pod rot disease under field conditions. The PGPR isolates NEG27, NWG12, and NEG3—were applied at three concentrations (0.5%, 1%, and 1.5%) either individually or in various combinations to assess their individual and synergistic effects on disease suppression. The bacterial formulations were prepared using talc powder as a carrier, with a final concentration of 2 × 10⁸ CFU/mL, and were applied to the aerial parts of the cocoa plants using battery operated Knapsack sprayer (Maya Fertilizer and Pesticides, India). In addition to PGPR treatments, a 0.3% copper oxychloride (COC 50% WP) fungicide treatment was included as a positive control, and a water-treated plot was maintained as a negative control. The experiment was laid out in a Randomized Block Design (RBD) with three replications, and ten trees per treatment per replication were selected for observations. Four foliar sprays were applied at 15-day intervals, and disease severity was assessed every 15 days using a 1–8 disease severity scale as described by Iwar et al. (1997) (Table 1). 

**Table 1:** Cocoa pod rot disease severity rating scale

**Rating**	**Disease Severity**
1	No symptom
2	1-5 localized lesions
3	6-15 localized lesion
4	>15 localized lesions
5	1-5 expanding lesions
6	6-15 expanding lesions
7	>15 expanding lesions
8	Coalesced lesions

 The per cent disease index (PDI) was calculated as per the formula given by Wheeler [38] and Area Under Disease Progress Curve (AUDPC) was assessed by using the formula given by Wilcoxson et al. [39].

$$\mathrm{PDI}\;=\frac{\mathrm{Sum}\;\mathrm{of}\;\mathrm{individual}\;\mathrm{disease}\;\mathrm{ratings}}{\mathrm{Number}\;\mathrm{of}\;\mathrm{observations}\;\mathrm{assessed}\;\mathrm x\;\mathrm{Maximum}\;\mathrm{Disease}\;\mathrm{rating}}\;\times\;100$$

$$\:\mathbf{A}\mathbf{U}\mathbf{D}\mathbf{P}\mathbf{C}={\sum\:}_{i=1}^{k}\frac{1}{2}\left({S}_{i}+{S}_{i-1}\right)\times\:d$$

Where,

Si = Disease incidence at i^th^ day of evaluation.

k = Number of successive evaluations of the disease.

d = Interval between i and i-1evaluation of disease.

To assess the yield, the pods were harvested separately and the bean weight is recorded. The average of all three replications each treatment was noted as total yield per treatment. Yield attributes such as pod length (cm), pod width (cm), wet cocoa beans (20 beans) weight (g), dry cocoa bean weight (20 beans) (g), pod weight (g) and Yield (kg)/tree werealso recorded. The cost of cultivation and gross returns per ha were calculated. The net returns per ha was calculated by deducting the total cost of cultivation from the total monetary value of the produce. To know the rate of return per rupee invested, benefit-cost ratio was determined by using the formula.

$$\mathrm B:\mathrm C\;=\;\frac{\mathrm{Gross}\;\mathrm{income}}{\mathrm{Total}\;\mathrm{cost}\;\mathrm{of}\;\mathrm{cultivation}\left(\mathrm{Rs}.\;\mathrm{ha}^{-1}\right)\;}$$

### Statistical analysis

Statistical analyses were conducted using SPSS (Version 17). The data sets in all the experiments were replicated 3–5 times. The per cent data was arcsine transformed before proceeding to analysis. Treatments were compared using ANOVA, applying the significant differences (SD) at a significance level of 5% (*p* ≤ 0.05).

## Results

### Biochemical characterization of bacterial isolates

**Fig. 1:**
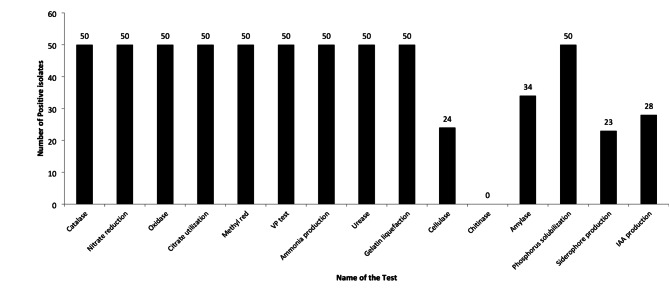
Biochemical profiling of PGPR isolates from cocoa rhizosphere

The biochemical characterization of the 50 rhizospheric bacterial isolates revealed uniformly positive results for multiple key metabolic traits. All isolates tested positive for catalase and oxidase activity, indicating robust aerobic metabolism and reactive oxygen species detoxification. They also showed positive responses for nitrate reduction, citrate utilization, methyl red, and Voges-Proskauer (VP) tests, suggesting metabolic versatility through both mixed-acid and butylene glycol fermentation pathways, as well as effective nitrogen assimilation. Additionally, all 50 isolates were positive for ammonia production, urease activity, and gelatin liquefaction, reflecting their roles in nitrogen mineralization, urea hydrolysis, and the degradation of complex organic substrates (Fig. 1). These traits collectively confirm the metabolic competence and rhizospheric adaptability of all 50 isolates, supporting their potential as effective Plant Growth-Promoting Rhizobacteria (PGPR). Among the 50 isolates, 24 (48%) showed cellulase activity on LB-CMC agar, confirmed by clear halos after Congo red staining. High cellulolytic activity was observed in NEG-27 (14 mm), NEG-14 (13 mm), and NWG-12 (12 mm), indicating potential in organic matter breakdown and rhizospheric colonization. NEG-16 and NEG-3 followed with 11 mm and 10 mm zones, respectively, while the remaining 26 isolates lacked cellulase activity. For amylase activity, 34 isolates (68%) produced halo zones (4–14 mm), indicating starch hydrolysis ability, which supports nutrient cycling and root colonization. None of the isolates showed chitinase activity, implying a lack of direct fungal cell wall degradation, and suggesting their biocontrol potential may rely on other mechanisms such as competition or induced resistance. All isolates (100%) exhibited phosphate solubilization on Pikovskaya’s agar, indicating their potential to mobilize insoluble phosphates. Additionally, 23 isolates (46%) produced siderophores, enhancing iron availability and possibly suppressing pathogens. IAA production was detected in 28 isolates (56%), supporting their role in promoting root growth and plant vigor. 

### Symptomatology, morphological characters and pathogenicity of *P. palmivora*

**Fig. 2:**
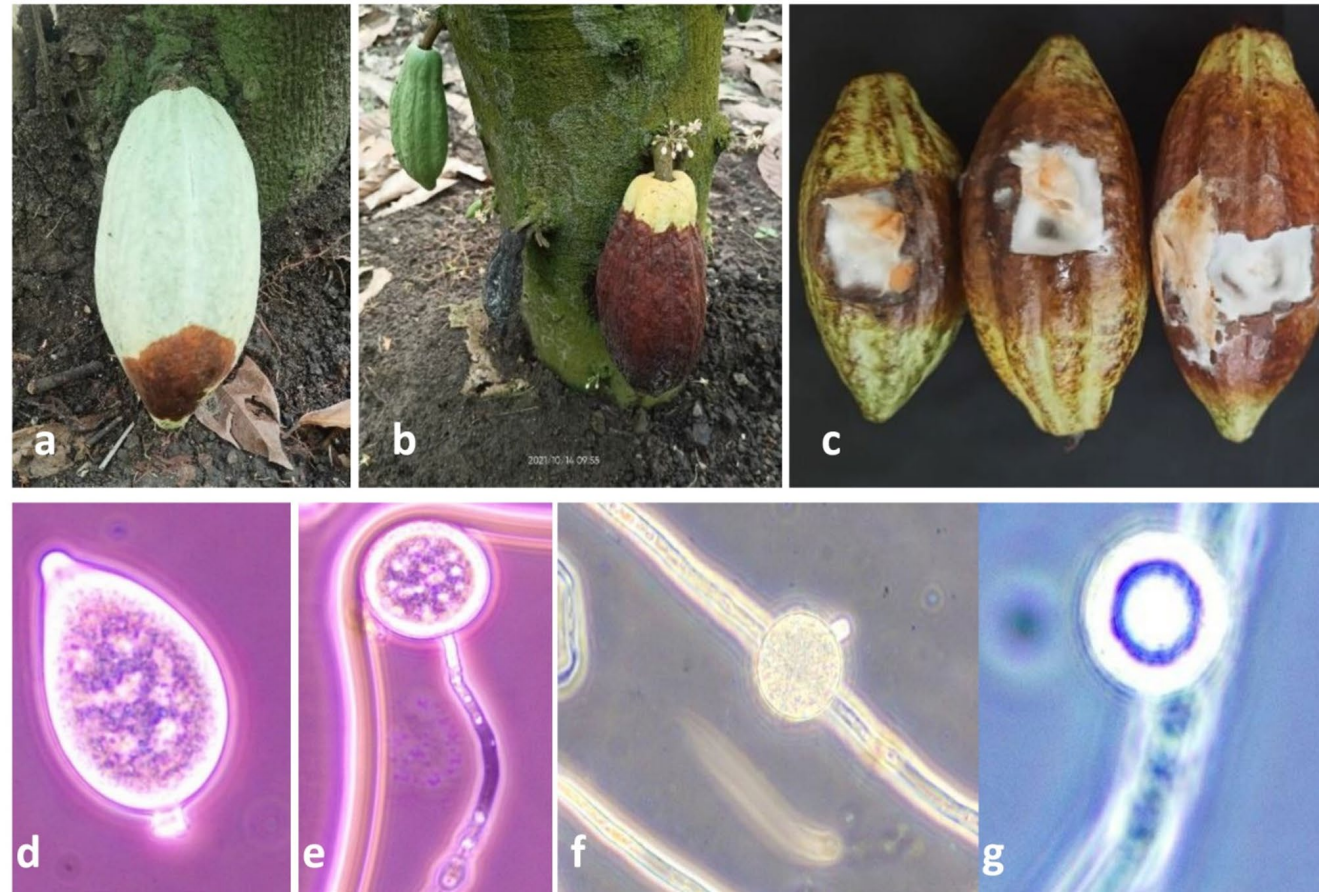
Symptoms of cocoa *Phytophthora* pod rot disease : chocolate brown spot (**a**) and complete pod rot (**b**); Symptoms of pathogenicity test on cocoa variety VTLCH-2 (**c**); morphological characters of the *Phytophthora sp.* : pear shaped papillate sporangia with caducous short pedicel (**d**); terminally globose chlamydospores (**e**); globose intercalary chlamydospore (**f**); thick walled chlamydospore (**g**)

Pod rot of cocoa begins as a chocolate-brown oval to round spot on the pod surface, which rapidly spreads to cover the entire pod. Infected pods become brown to black, invading the pod interior and affecting the beans eventually becoming mummified, ultimately emitting a characteristic fishy odour (Fig. 2a & b). Using the IDPhy database as a reference, the morphological characteristics were assessed, sporangia were pear-shaped, papillate with caducous short pedicels. Chlamydospores were globose shape, produced terminally and intercalary with thick walls (Fig. 2d, e, f, & g). After cultural and morphological confirmation, a pathogenicity test was performed on cocoa variety VTLCH-2. The test exhibited typical characteristic symptoms of pod rot, starting with brown spots on the pod surface accompanied by white mycelial growth. These spots rapidly expanded, eventually covered the entire pod surface. The internal beans were also severely affected, showing discoloration and decay **(**Fig. 2c**)**. The infection was later confirmed by re-isolation of the pathogen and microscopic and molecular studies.

### Molecular detection and phylogeny

**Fig. 3:**
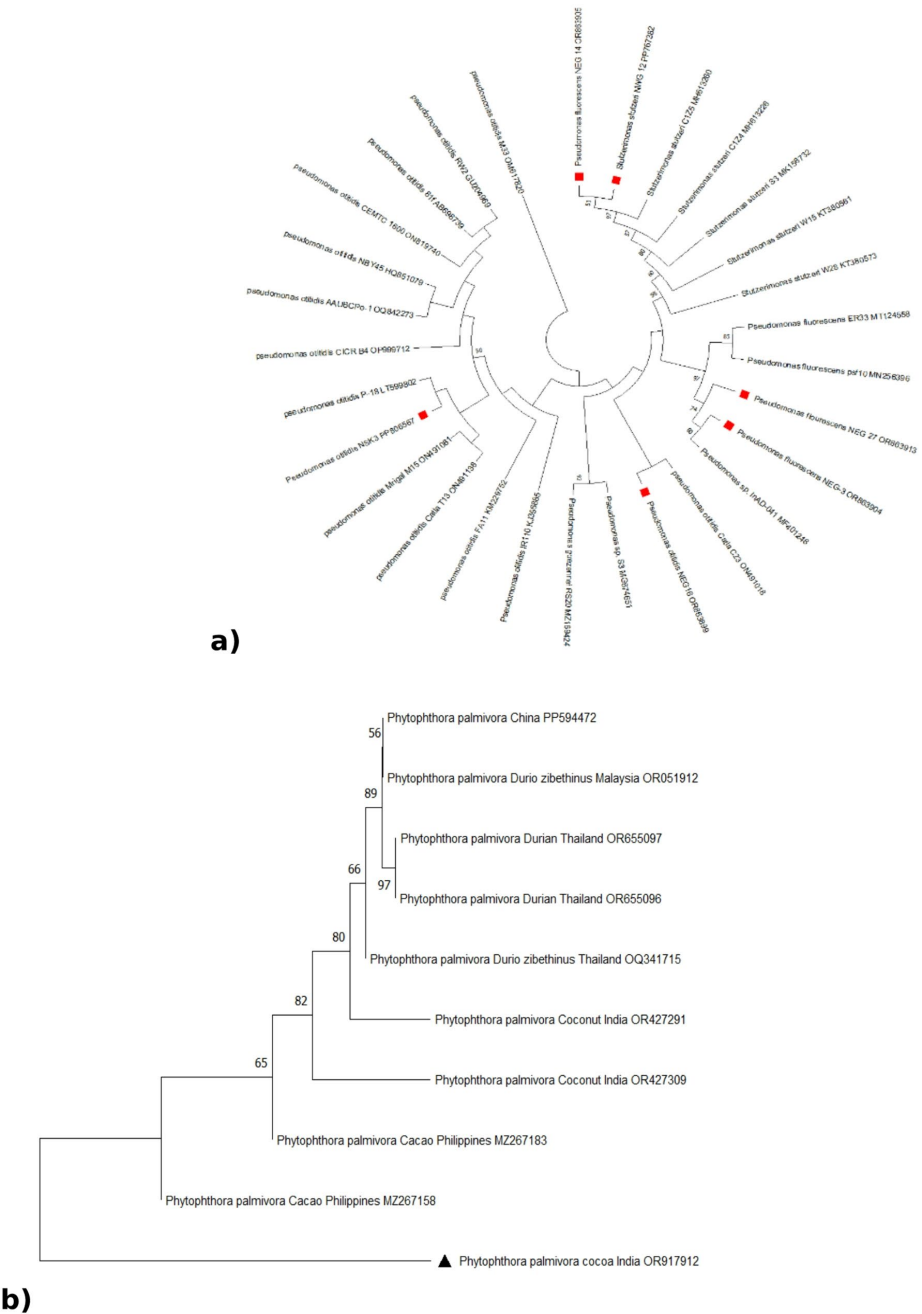
Phylogenetic tree constructed based on bacterial 16SrRNA sequences using Maximum Likelihood method with 1000 bootstrap values (square marked are isolates used in the present study) (**a**); Phylogenetic tree constructed based on ITS sequences of *P. palmivora* using Maximum Likelihood method with 1000 bootstrap values (triangular marked are isolate used in the present study) (**b**)

The oligonucleotide primers targeted for 16 S rRNA genes of bacterial isolates resulted in amplicons of 1.5 kb size. The nucleotide sequences partial 16 S rRNA of six isolates when subjected to phylogenetic analysis (Fig. 3a) along with available corresponding sequences in NCBI database revealed, isolates NEG27 (OR863913) and NEG3 (OR863904) recognised as *Pseudomonas fluorescens*, with 99.06% and 99.29% similarity whereas the isolate NWG12 (PP767382) as *Stutzerimonas stutzeri* (= *Pseudomonas stutzeri*) with 99.08% similarity, the isolates NEG14 (OR863905), NEG16 (OR863899) and NSK3 (PP806567) were recognized as *Pseudomonas otitidis* with 100% identity. For fungal pathogen detection, oligonucleotide primers targeting 3 regions such as ITS, β-tubulin and COX-II resulted in amplicon sizes of 900 bp, 450 bp and 1250 bp respectively. The pathogen was amplified for all the three regions and ITS region was sequenced. The sequence was deposited in the GenBank data-base and the obtained accession number was OR917912. Phylogenetic analysis (Fig. 3b) of the study isolate along with closely related corresponding sequences in NCBI database revealed close match with *Phytophthora palmivora* isolate from Philippines (MZ267158).

### *In-vitro* antagonism of bacterial isolates against *P. palmivora*

**Fig. 4:**
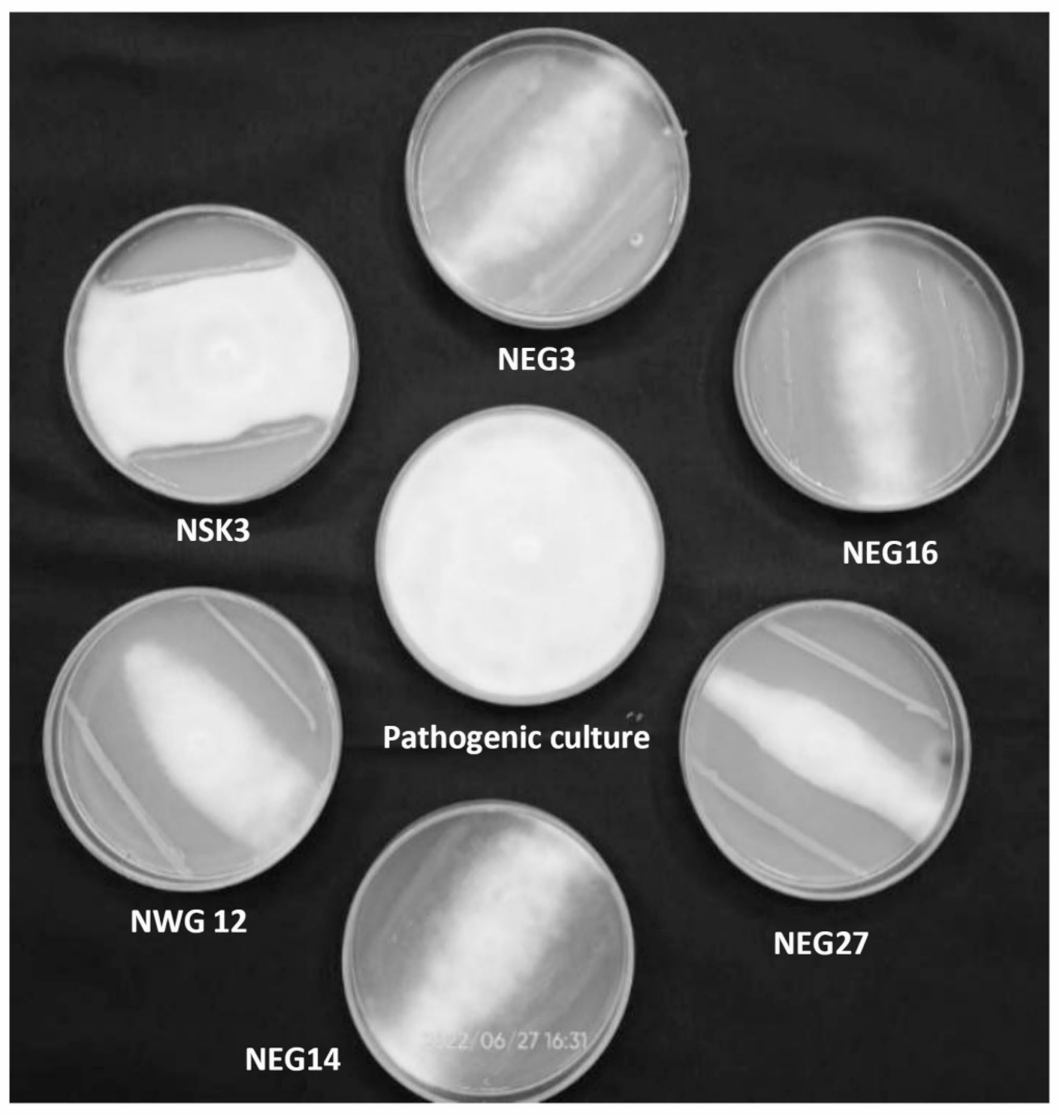
Antagonistic effect of six potential bacterial isolates against *P. palmivora* in dual-culture plate technique

**Fig. 5:**
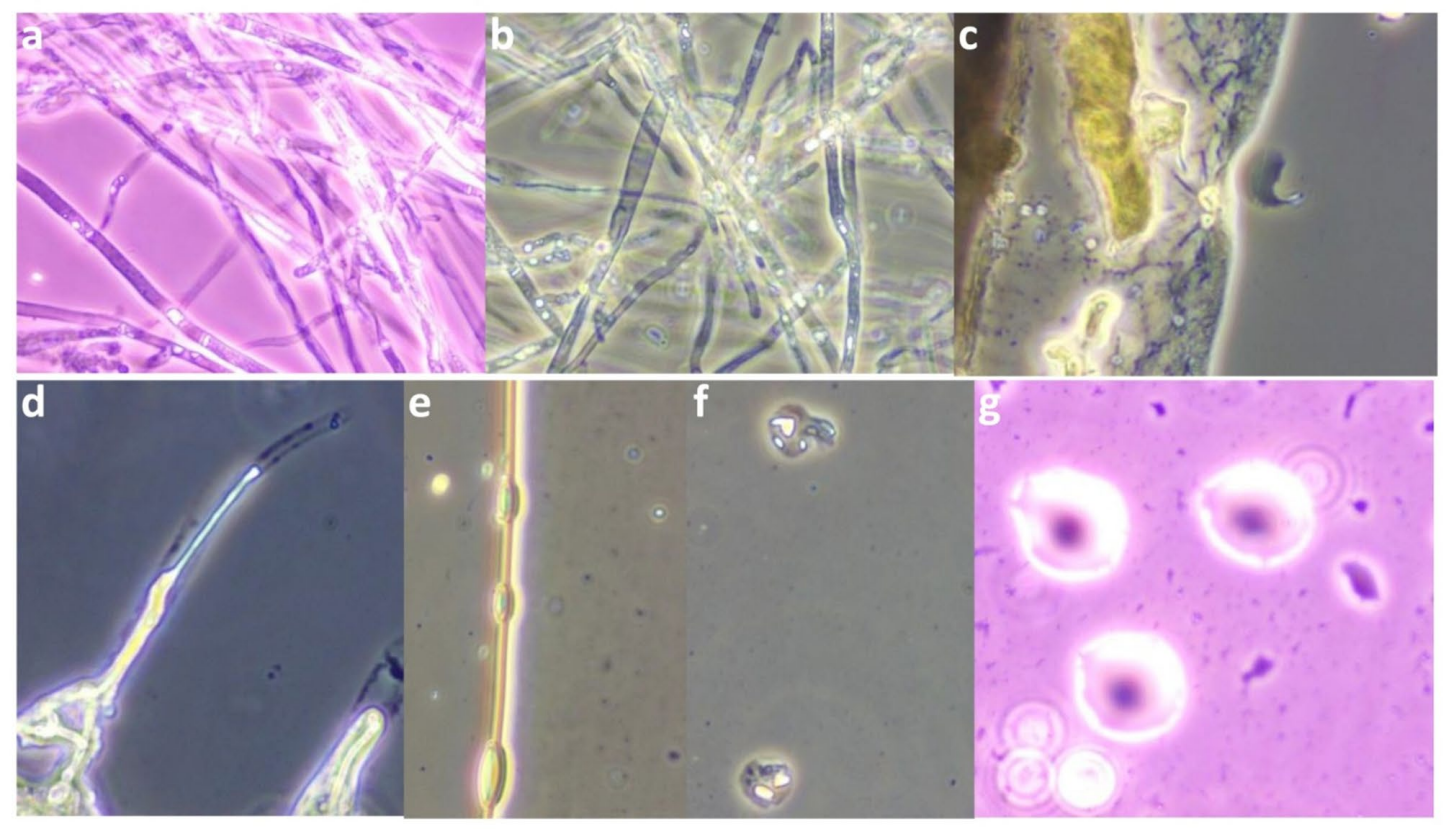
Microscopic view of PGPR isolates on *P. Palmivora* vegetative and reproductive structure at zone of interaction: Hyphal disintegration (**a**);hyphal twisting (**b**); hyphal shrinking (**c**); hyphal tip disintegration (**d**); hyphal bulging (**e**); misshapen zoospores in the sporangia (**f**); lack of zoospores differentiation in the sporangia (**g**)

Among the fifty rhizobacterial isolates, NEG27 was found to be significantly superior PGPR with maximum inhibition (82.26%) *P. palmivora*, followed by NWG12 with an inhibition percentage of 77.41%, which was on par with NEG14 at 76.30% inhibition (Table 2). Continued incubation up to four days after inoculation (DAI) revealed that only nine bacterial isolates NEG27, NWG16, NEG14, NEG9, NWG12, NSK3, NWG14, NEG3 and NEG6 produced characteristic zones of inhibition against *P. palmivora* (Fig. 4). Distinct morphological alterations in *P. palmivora* hyphae were observed during dual-culture with the rhizobacterial isolates (Fig. 5), including hyphal tip disintegration, protoplasm disintegration, mis shapen zoospores in sporangia, lack of zoospore differentiation within sporangia, hyphal bulging, twisting and shrinking.

**Table 2:** Antagonistic efficacy of superior bacterial isolates on inhibition of *P. palmivora* radial growth in dual culture assay

S. No.	Isolate	1 DAI			2 DAI			3 DAI			4 DAI		
		RG (cm)	I (%)	ZI (cm)	RG (cm)	I (%)	ZI (cm)	RG (cm)	I (%)	ZI (cm)	RG (cm)	I (%)	ZI (cm)
1	NEG3	2.20	12.00 (20.27)	2.80	4.70	34.36 (35.88)	0.30	4.90	42.35 (35.88)	0.10	2.68	**68.59 (55.94)**	2.32
2	NEG14	1.50	40.00 (39.23)	3.50	4.67	34.88 (36.20)	0.33	4.67	45.10 (36.19)	0.33	2.13	76.30 (60.87)	2.87
3	NEG16	1.40	44.00 (41.54)	3.60	4.83	32.56 (34.79)	0.17	4.83	43.14 (34.79)	0.17	2.97	43.70 (41.38)	
4	NEG27	0.70	72.00 (58.05)	4.30	4.32	39.72 (39.07)	0.68	4.32	49.18 (39.06)	0.68	1.60	**82.26 (65.09)**	3.40
5	NWG12	1.40	44.00 (41.55)	3.60	4.43	38.13 (38.13)	0.57	4.43	47.84 (38.13)	0.57	2.03	**77.41 (61.62)**	2.97
6	NSK3	1.70	32.00 (34.45)	3.30	4.53	36.74 (37.31)	0.47	4.53	46.67 (37.31)	0.47	3.60	**59.96 (50.75)**	1.40
7	*P.palmivora* control	2.50	-	-	7.17	-	-	8.40	-	-	8.94	-	-
SEM ±		0.04	0.80		0.04	1.60		0.95	1.42		0.11	0.85	
CD (*p* ≤ 0.05)		0.11	2.24		0.12	4.48		2.95	4.58		0.31	2.40	
CV (%)		3.60	5.81		2.36	11.6		4.25	8.99		3.00	4.89	

Values in parenthesis are arc sine transformation values

Each treatment replicated thrice

*RG* radial growth, *I*-inhibition, *ZI-*zone of inhibition

### Rhizobacterial treatment on germination and vigour index in cocoa seeds

The application of PGPR antagonists significantly influenced the germination percentage of cocoa seeds (var. VTLCH2), which ranged from 64.44 to 84.44% across treatments. The highest germination rate (84.44%) was observed in seeds treated with isolate NEG27, followed by NEG3 (80%), both of which were significantly superior to the untreated control (62.22%). Seedling growth parameters, including shoot and root length, were evaluated 20 days after sowing. Significantly the highest shoot length (17.05 cm) and root length (18.22 cm) were recorded in seedlings treated with NEG27, identified as *P. fluorescens*. This was followed by NWG12 for shoot length (14.32 cm) and both NWG12 and NEG3 for root length (12.33 cm each). The lowest shoot (4.25 cm) and root lengths (4.22 cm) were recorded in untreated control seedlings. Correspondingly, the seedling vigour index (SVI) showed significant enhancement in PGPR-treated seeds, ranging from 3056.73 (NEG27) to 624.00 (NSK3), compared to the untreated control (564.67) (Table 3). These results indicate the strong potential of certain PGPR isolates, particularly NEG27 and NEG3, in enhancing cocoa seed germination and early seedling vigor.

**Table 3:** Effect of promising rhizobacterial treatment on germination and vigour index in cocoa seeds

Tr. No.	Treatments (ST @ 10^8^ CFU/ml)	Germination per cent (%)	Shoot length (cm)	Root length (cm)	Seedling length (cm)	Vigour index
T_1_	NEG27	84.44	17.05	18.22	35.27	3056.73
T_2_	NWG12	77.78	14.32	12.33	26.65	2132.00
T_**3**_	**NEG3**	**80.00**	**12.25**	**12.33**	**24.58**	**1966.40**
T_4_	NEG16	64.44	6.53	5.32	11.85	790.00
T_5_	NEG14	71.11	8.95	9.95	18.9	1386.00
T_6_	NSK3	64.44	5.23	4.13	9.36	624.00
T_7_	Check	62.22	4.25	4.22	8.47	564.67
	**SEM ±**	2.06	0.40	0.29		
	(*p* ≤ 0.05)	6.24	1.21	0.87		
	**CV (%)**	4.90	7.30	5.20		

*ST* -Seed treatment

NEG27 = *P. fluorescens*, NEG3 = *P. fluorescens*, NWG12 = *S. stutzeri (P. stutzeri)*, NEG16 = *P. otitidis*, NEG14 = *P. otitidis* and NSK3 = *P. otitidis *

^*^Values are mean of three replications

### Field testing of promising PGPR isolates on pod rot disease suppression, AUDPC and yield of cocoa

**Fig. 6:**
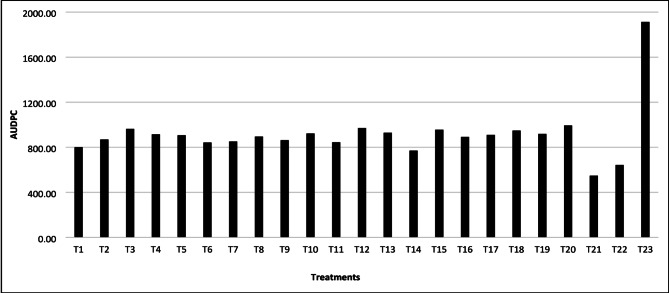
Effect of antagonistic PGPR bacterial isolates on pooled cocoa pod rot disease AUDPC under field conditions during 2022-23 and 2023-24

Three PGPR isolates, NEG27 (*Pseudomonas fluorescens*), NWG12 (*Stenotrophomonas stutzeri*), and NEG3 (*P. fluorescens*) were selected based on their superior PGPR traits and effects on seed germination and seedling vigor. Among the treatments tested against *P. palmivora*, the combined application three PGPR isolates at 1.5% concentration (T21) significantly reduced disease severity and and its efficiency was on par with the standard 0.3% copper oxychloride treatment. There is significant different in the disease supression; The treatment T21: AUDPC 548.10 vs. control 1912.90 indicating effective disease suppression (Fig. 6). In terms of productivity, T21 recorded the highest mean pod weight of 550.62 g on par with COC treatment (552.25 g). Similarly, wet bean weight (20 beans) was 43.85 g in T21, nearly equal to 44.25 g in COC-treated plants, and dry bean weight (20 beans) in T21 was 22.12 g, on par with the COC treatment. Significantly, T21 achieved the highest yield among PGPR treatments at 3275 kg/ha, closely matching the COC-treated plot (3460 kg/ha) and substantially outperforming the untreated control (1735 kg/ha). The Benefit-Cost Ratio (BCR) ranged from 3.59 to 7.06 across treatments, with T21 achieving 6.7, second only to the COC treatment (7.06), highlighting its economic feasibility as a sustainable biocontrol alternative (Table 4).

**Table 4:** Effect different concentrations and combinations of PGPR on cocoa *Phytophthora* pod rot disease severity

Sl. No.	Treatments	Per cent Disease Index (PDI)				
		Day before first spray	15 days after First spray (15DAT)	15 days after Second spray (30 DAT)	15 days after Third spray (45 DAT)	15 days after Fourth spray (60 DAT)
T_1_	0.5% NEG27	13.43(21.50)	13.43 (21.50)	13.20 (21.44)	11.60 (19.86)	10.50 (18.90)
T_2_	0.5% NWG12	17.23 (24.51)	16.25 (23.77)	16.03 (23.60)	15.15 (22.91)	17.15 (24.46)
T_3_	0.5%NEG3	18.03 (25.13)	18.39 (25.44)	17.43 (24.65)	16.76 (24.14)	17.76 (24.92)
T_4_	T_1_ + T_2_	16.97 (24.32)	15.26 (22.99)	14.59 (22.45)	12.27 (20.50)	12.27 (20.50)
T_5_	T_1_ + T_3_	18.33 (25.35)	16.30 (23.81)	15.57 (23.24)	15.20 (23.12)	14.43 (22.34)
T_6_	T_2_ + T_3_	16.80 (23.08)	15.57 (23.24)	14.62 (22.50)	13.52 (21.62)	11.57 (19.87)
T_7_	T1 + T2 + T3	17.50 (24.72)	15.06 (22.66)	14.59 (22.45)	13.20 (21.44)	12.20 (20.44)
T_8_	1% NEG27	15.43 (23.12)	15.23 (23.10)	14.59 (22.45)	13.02 (21.56)	12.80 (21.10)
T_9_	1% NWG12	16.60 (24.02)	15.57 (23.24)	15.57 (23.24)	14.47 (22.34)	14.67 (22.51)
T_10_	1% NEG3	24.10 (29.38)	23.25 (28.79)	21.94 (27.86)	20.38 (26.70)	20.22 (26.57)
T_11_	T_8_ + T_9_	15.57 (23.20)	14.61 (22.46)	13.43 (21.50)	10.47 (18.83)	11.63 (19.93)
T_12_	T_9_ + T_10_	17.73 (24.90)	16.90 (24.27)	15.73 (23.36)	14.73 (22.25)	13.73 (21.75)
T_13_	T_8+_T_10_	18.33 (25.35)	16.30 (23.81)	15.63 (23.28)	14.63 (22.48)	13.53 (21.55)
T_14_	T_8_ + T_9+_T_10_	13.27 (21.36)	12.83 (20.99)	10.33 (18.69)	8.67 (17.12)	7.52 (15.91)
T_15_	1.5% NEG27	20.63 (27.01)	20.33 (26.80)	19.33 (26.08)	17.01 (24.33)	17.34 (24.59)
T_16_	1.5%NWG12	17.53 (24.71)	19.12 (25.93)	18.45 (25.44)	17.20 (24.50)	17.53 (24.74)
T_17_	1.5% NEG3	23.27 (28.82)	22.50 (28.31)	21.17 (27.39)	19.33 (26.08)	19.50 (26.20)
T_18_	T_15_ + T_16_	19.67 (26.32)	19.50 (26.20)	18.73 (25.64)	16.42 (23.90)	15.75 (23.37)
T_19_	T_16_ + T_17_	21.40 (27.54)	19.17 (25.96)	17.03 (24.37)	15.28 (23.01)	14.61 (22.46)
T_20_	T_15_ + T_17_	20.03 (26.58)	18.50 (25.47)	17.42 (24.66)	16.41 (23.88)	15.74 (23.38)
T_21_	T_15_ + T_16_ + T_17_	14.20 (22.12)	11.93 (20.21)	9.27 (17.72)	5.27 (13.26)	3.40 (10.61)
T_22_	0.3% COC	16.63 (24.06)	12.97 (21.10)	9.30 (17.76)	6.33 (14.58)	4.60 (12.37)
T_23_	Water	24.73 (29.68)	26.65 (31.08)	28.72 (32.41)	33.52 (35.58)	40.59 (39.58)
SEm±		1.21	0.92	1.02	0.98	0.98
CD (*p* ≤ 0.05)		NS	2.61	2.91	2.78	2.78
CV (%)		9.20	9.70	11.70	12.30	12.10

Values are mean of three replications

Values in parenthesis are arc sine transformed

*DAT* Days after treatment

## Discussion

PGPR colonizing the surface or interior of roots play pivotal roles in enhancing plant growth and development, both directly and indirectly [40, 41]. Recent studies have emphasized their contributions to plant health through mechanisms such as nutrient uptake facilitation, production of growth-promoting substances like phytohormones, and enhancement of stress tolerance [17, 42, 43]. In this study, 50 PGPR were isolated from coconut and oil palm-based cropping systems of cocoa. Among these, six isolates- *P. fluorescens* (NEG27 and NEG3), *S. stutzeri* (NWG12) and *P. otitidis* (NEG14, NEG16, and NSK3) demonstrated promising plant growth-promoting traits. All isolates tested positive for catalase, nitrate reduction, oxidase, citrate utilization, methyl red, Voges-Proskauer, ammonia production, urease, and gelatin liquefaction tests under laboratory conditions. These findings are in accordance with previous studies showing that *P. stutzeri* and *P. fluorescens* are catalase-positive [44, 45]. *Pseudomonas* species, frequently reported as PGPR [33, 46, 47], are renowned for their metabolic diversity and physiological traits that contribute to ecological success and agricultural applications. They exhibit robust nitrate reduction abilities, utilizing nitrate as a terminal electron acceptor under anaerobic conditions. Oxidase activity, indicative of cytochrome c oxidase presence is another characteristic trait. Additionally, citrate utilization highlights their adaptability to using citrate as a sole carbon source. Positive results in methyl red and Voges-Proskauer tests indicate their ability to perform mixed acid fermentation and produce acetoin from glucose fermentation. These bacteria also demonstrate ammonia production, urease activity, and gelatinase activity, reflecting their metabolic versatility [44]. Such traits underscore their ecological significance and potential in sustainable agriculture [17, 42, 43].

Treatment of cocoa seeds with selected PGPR isolates led to a marked improvement in seedling emergence and early growth. This observation aligns with previous studies, including Hardiansyah et al., [48], who found that PGPR enhanced seed germination and vigor by suppressing seed-borne mycoflora. Similarly, Rajeela et al. [49] reported that *Pseudomonas putida* KDSF23, a cocoa-derived isolate, significantly boosted plant growth and outperformed conventional low-volume fertilizer application, underscoring its potential as a PGPR. Ferrás-Negrín and Bustamante-González et al., [50] further supported these findings by showing that inoculation with *Pseudomonas* spp. increased seed emergence and early seedling development in cocoa by up to 38.9%. PGPR inoculation has been linked to increased amylase activity during germination, facilitating starch hydrolysis into metabolizable sugars for root and shoots growth [51]. Moreover, phytohormone production, particularly IAA, plays a crucial role in promoting root development and nutrient uptake [52]. In this study, all selected isolates demonstrated IAA production, with some exhibiting higher levels than previously reported strains [53–55].

Plant Growth-Promoting Rhizobacteria (PGPR) isolates has demonstrated significant antagonistic activity against *P. palmivora*, the causative agent of black pod disease in cocoa. In vitro assays revealed that *P. fluorescens* (NEG27) showing the highest suppression. Supporting these findings, Thomas et al. confirmed the antagonistic potential of fluorescent *Pseudomonas* spp. isolated from cocoa roots, identifying them as strong candidates for biocontrol applications. *Pseudomonas chlororaphis* strain CP07 also showed high efficacy, significantly reducing disease severity across various *Theobroma cacao* genotypes, as reported by Acebo-Guerrero et al. [8]. In a separate study, Alsultan et al. [56] highlighted *P. aeruginosa* as an effective endophytic antagonist of *P. palmivora* in Malaysia. Consistent antagonism by *P. chlororaphis* in cacao rhizospheres further supports its role in integrated disease management strategies. In addition to *Pseudomonas* species, both *P. fluorescens* and *Bacillus subtilis* exhibited inhibitory effects on *P. palmivora*, with *B. subtilis* producing the largest inhibition zones, according to Pratama et al. [18]. Field trials by Larbi-Koranteng et al. [57] showed that combinations of two or more bioagents have effectively reduce black pod incidence by 40–67%. This highlights the potential for synergistic effects when using combined, which can increase efficacy compared to individual isolates.

Plant Growth-Promoting Rhizobacteria (PGPR) utilize diverse mechanisms to suppress *Phytophthora* spp., making them effective biocontrol agents. One primary mode of action is the production of secondary metabolites, such as phenazines and cyclic lipopeptides, which disrupt the pathogen’s cell membranes, thereby inhibiting its growth and viability [58]. Beyond direct antagonism, PGPR also activate Induced Systemic Resistance (ISR) in host plants, enhancing their defensive capacity against pathogens like *Phytophthora* [59, 60]. Foliar applications of PGPR strains have been shown to significantly reduce the severity of Phytophthora pod rot in cocoa. Notably, *P. fluorescens* and *S. stutzeri* have emerged as potent antagonists, corroborating earlier reports of their effectiveness against soil-borne pathogens [18, 54, 55, 58, 61]. Antibiosis—another key mechanism—involves the secretion of antibiotics and lytic enzymes that degrade pathogen cell structures. These bioagents have been observed to induce significant morphological damage in *P. palmivora*, including hyphal membrane invagination, cell wall disintegration, and cytoplasmic breakdown [62–64]. Together, these multifaceted actions underscore the promising role of PGPR in integrated disease management strategies for cocoa cultivation.

## Conclusions

This study demonstrated that PGPR isolates significantly reduced disease severity, improved plant growth, and enhanced yield-related parameters in cocoa. Among the 50 bacterial isolates, six indigenous PGPR strains, *Pseudomonas fluorescens* (NEG27 and NEG3), *Stutzerimonasstutzeri* (NWG12) and *Pseudomonas otitidis* (NEG14, NEG16, and NSK3) exhibited diverse plant growth-promoting traits and antifungal activity, effectively controlling *Phytophthora* pod rot. These findings highlight their potential as biofertilizers and biocontrol agents in sustainable agriculture. However, transitioning from laboratory to field application requires rigorous greenhouse experiments and field trials to optimize formulations for consistent performance. Ongoing research focuses on developing stable integrated bio-formulations for commercialization and deployment in horticulture. 

## Data Availability

The DNA sequences generated during this study have been deposited in the NCBI GenBank repository. Accession numbers are OR863913, OR863904, PP767382, OR863905, OR863899 and PP806567 for PGPR and OR917912 for the pathogen.
